# Benefits and harms of mammography screening

**DOI:** 10.1186/s13058-015-0525-z

**Published:** 2015-05-01

**Authors:** Magnus Løberg, Mette Lise Lousdal, Michael Bretthauer, Mette Kalager

**Affiliations:** Institute of Health and Society, University of Oslo, N-0317 Oslo, Norway; Department of Transplantation Medicine, Oslo University Hospital, 0424 Oslo, Norway; Department of Epidemiology, Harvard School of Public Health, Boston, MA 02115 USA; Department of Public Health, Aarhus University, 8000 Aarhus C, Denmark; Department of Medicine, Sorlandet Hospital, 4604 Kristiansand, Norway; Telemark Hospital, 3710 Skien, Norway

## Abstract

Mammography screening for breast cancer is widely available in many countries. Initially praised as a universal achievement to improve women's health and to reduce the burden of breast cancer, the benefits and harms of mammography screening have been debated heatedly in the past years. This review discusses the benefits and harms of mammography screening in light of findings from randomized trials and from more recent observational studies performed in the era of modern diagnostics and treatment. The main benefit of mammography screening is reduction of breast-cancer related death. Relative reductions vary from about 15 to 25% in randomized trials to more recent estimates of 13 to 17% in meta-analyses of observational studies. Using UK population data of 2007, for 1,000 women invited to biennial mammography screening for 20 years from age 50, 2 to 3 women are prevented from dying of breast cancer. All-cause mortality is unchanged. Overdiagnosis of breast cancer is the main harm of mammography screening. Based on recent estimates from the United States, the relative amount of overdiagnosis (including ductal carcinoma *in situ* and invasive cancer) is 31%. This results in 15 women overdiagnosed for every 1,000 women invited to biennial mammography screening for 20 years from age 50. Women should be unpassionately informed about the benefits and harms of mammography screening using absolute effect sizes in a comprehensible fashion. In an era of limited health care resources, screening services need to be scrutinized and compared with each other with regard to effectiveness, cost-effectiveness and harms.

## Introduction

The verb 'to screen' is defined as 'to sift by passing through a screen' [1]. 'To 'sift'; derives from an old Dutch word ('zeef'); a 'utensil consisting of a circular frame with a finely meshed or perforated bottom, used to separate the coarser from the finer particles of any loose material' [1].

The definitions of screening vary among different cultures, settings, and time periods [2,3]. In general, all definitions of screening include an identification of disease or disease precursor among presumptively healthy individuals. There are mainly two different approaches of cancer screening: prevention of disease by finding and removing premalignant precursors of cancer; and early detection of cancer where the goal is to treat the invasive cancer in an early curable stage [4]. In 1968, the World Health Organization suggested 10 principles that should be fulfilled before implementing screening in a population (Table 1) [5]. Some of the principles regard knowledge about biologic development of cancer (principles 4 and 7).

**Table 1:** **The World Health Organization’s 10 principles of screening**

1.	The condition sought should be an important health problem
2.	There should be an accepted treatment for patients with recognized disease
3.	Facilities for diagnosis and treatment should be available
4.	There should be a recognizable latent or early symptomatic stage
5.	There should be a suitable test or examination
6.	The test should be acceptable to the population
7.	The natural history of the condition, including development from latent to declared disease, should be adequately understood
8.	There should be an agreed policy on whom to treat as patients
9.	The cost of case-findings (including diagnosis and treatment of patients diagnosed) should be economically balanced in relation to possible expenditure on medical care as a whole
10.	Case finding should be a continuing process and not a 'once and for all' project

Screening for breast cancer with mammography aims at detecting breast cancer at an early, curable stage. For early detection by screening to be beneficial, we anticipate a continuous, linear growth pattern of tumors, and that breast cancer has not spread at the time when tumors are detectable at mammography. Thus, if the assumptions of tumor growth are not correct or if growth of tumors is heterogenic, screening mammography might not be an adequate tool to reduce the burden of breast cancer [6].

The idea of early detection started in the US in the early 20^th^ century with educational mass campaigns where the message of 'do not delay' seeking medical help for a variety of cancer signs and symptoms was central [7]. However, none of these early campaigns had an effect on the mortality of breast cancer [8]. In 1963 the first randomized trial of mammography screening was launched within the Health Insurance Plan in New York [8], and several other trials followed [9]. Most of the trials were performed before widespread use of anti-estrogens and modern chemotherapy with the exception of the Canadian National Breast Screening Study and the age trial [10,11].

In contrast to other cancer screening tools, mammography screening was evaluated in randomized trials before it was widely recommended and implemented. Nevertheless, there has been a continuous discussion of mammography screening, which started in full in 2000 after a Cochrane review of the randomized trials indicated little effect of screening [12]. More recently, the effect of mammography screening outside the experimental setting, in the modern era with improvements in awareness, diagnostics, and treatment, has been discussed [13,14].

The mammography debate has not only been about the beneficial effects of mammography screening, but more recently also the harms. In the last 10 years increasing awareness of overdiagnosis in mammography screening has emerged. Overdiagnosis is defined as the detection of tumors at screening that might never have progressed to become symptomatic or life-threatening in the absence of screening. This is a direct harm of screening because markers to distinguish the overdiagnosed tumors from the potential life-threatening tumors are lacking and, thus, all tumors are treated. Women with overdiagnosed tumors only experience the harms and side effects of treatment, without any benefit. In this review we discuss the benefits and harms of mammography screening and give an overview of the findings from randomized trials and from more recent observational studies from the era of modern diagnostics and treatment. We aim at presenting the benefits and harms per 1,000 women invited to mammography screening who started screening at age 50 years and were screened every second year until age 69 years; screening of this age group has been shown to achieve most of the benefit with less harm [15,16].

## Screening mammography

### Attendance rates

Mammography screening is recommended (and in Europe offered through organized programs) in most Western countries. However, in Switzerland an independent panel of experts (the Swiss Medical Board) reviewed the evidence on mammography screening and concluded that harms outweighed the benefits and recommended against mammography screening [17]; that is, that screening programs should not be implemented in areas where such programs do not exist and that the ongoing programs should be phased out. When screening is recommended, the eligible age range differs in different countries from 40 to 74 years [4,18,19]. The recommended interval between two screens varies from 1 to 3 years [18]. Mammography screening is well accepted; on average, more than half of eligible women attend screening mammography. In most countries, attendance rates are higher than 70%. Women aged 50 to 69 years have the highest attendance rate [18,19]. The attendance rate varies between countries (19.4% to 88.9%), and in different age groups. Most women who have participated once continue to participate.

### False positive tests

As with every diagnostic test the sensitivity and specificity of mammography screening are not perfect; various levels of sensitivity and specificity for detecting breast cancer have been published [20,21]. The risk of experiencing a false positive mammogram for women undergoing biennial screening from age 50 to 69 years in Europe is about 20% [21], and the risk of experiencing a biopsy due to a false positive test is 3% [21]. Based on data from the UK, 2.3% of all women with a false positive test had a lumpectomy, representing 76 out of 100,000 women screened in one screening round [22]. The risk is even higher in the US, where the 10-year false positive rate is 30%, and 50% of all women will experience a false positive mammogram at one time [23,24]. The challenges with a false positive test, apart from the monetary costs, are impaired psychological well-being and changes in health behavior among women with the false positive test. After 6 months, only 64% of those recalled due to a false positive test were declared cancer-free; after 1 year approximately 90% were declared cancer-free, and only after 2 years were all those who were in fact free of cancer declared cancer-free [25]. Research has shown that false positive results negatively influence women's psychological well-being during the period immediately after the tests, and a recent study showed that women with false positive findings experience psychological harm for at least 3 years after screening [26]. Women with false positive findings had higher use of health care services; 55% of women who experienced a positive recall returned to the outpatient clinic in the first year after screening, some up to eight times [27], and reported lower quality of life than those without [27,28]. Some women may also have altered health behavior and trust in the health care system [28].

### False negative tests

Interval cancers are cancers detected after a normal screening mammogram and before the next scheduled mammogram. Interval cancers either were overlooked at the last mammogram or are rapidly growing cancers that become apparent in the screening interval [29]. In a re-interpretation of interval cancers, around 35% were overlooked [30], while 65% were not visible at the latest mammogram and appeared in the interval between screening mammograms. Of all breast cancers detected among women who participate in screening, 28 to 33% are interval cancers [20], and this proportion seems to be stable in the different screening rounds [29]. Use of digital mammography is increasing, and detection rates of ductal carcinoma *in situ* (DCIS) and invasive cancers are higher. Whether this will decrease the proportion of interval cancers is unknown, but the rate of missed cancers seems to be similar to that of analogue, screen-film mammography [31]. One might anticipate, therefore, that the proportion of interval cancers with digital mammography will be comparable to that with analogue screen-film mammography. However, the increasing detection rates with digital mammography might increase the amount of overdiagnosis.

Women diagnosed with interval cancer do not benefit from early detection, but could be falsely reassured by their last normal mammogram and delay seeking medical care. However, this might not seem to be the case as women with interval cancer do not have poorer prognosis than women who chose not to utilize mammography screening [29].

**Figure 1:**
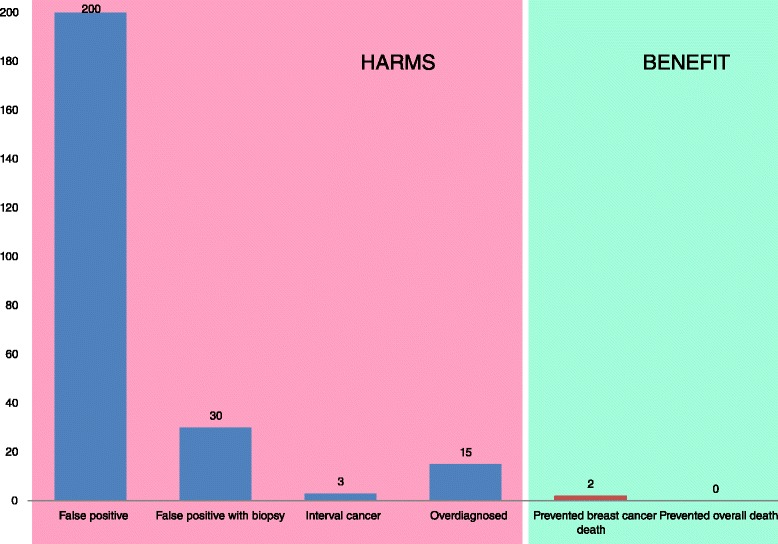
**Summary of benefits and harms when 1,000 women are screened every second years for 20 years starting at age 50.** Number of women with false positive mammograms and false positive biopsies are based on a review [32]. Number of interval cancers are based on reported number of interval cancer in the National Health Service breast screening programme [33]. The numbers of overdiagnosed and prevented breast cancer deaths are estimated based on 31% overdiagnosis [19] and 13 to 17% reduction in mortality from breast cancer [35]. These relative numbers are applied to the observed incidence of invasive breast cancer (women aged 50 to 69 years) and mortality (women aged 55 to 74 years) in the UK in 2007 [32]; this resulted in 15 overdiagnosed women and 2 to 3 prevented breast cancer deaths per 1,000 women. No deaths are prevented overall [9].

For 1,000 women invited to mammography screening every second year for 20 years from age 50, 200 will experience a false positive mammogram, 30 will undergo a biopsy due to a false positive mammogram, and 3 will be diagnosed with interval cancer [32,33] (Figure 1).

## Overdiagnosis

**Figure 2:**
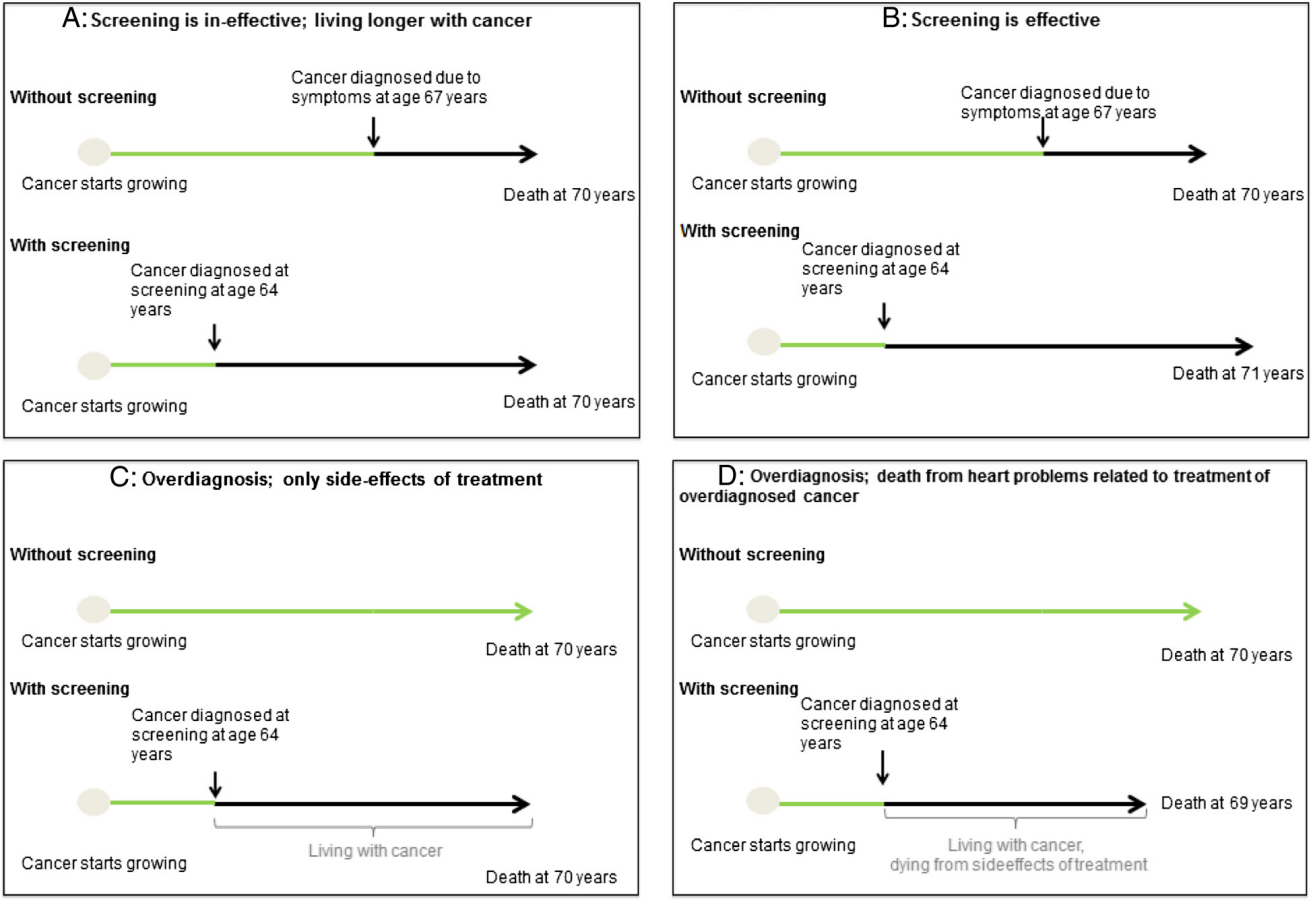
**Scenarios for different outcomes of screening mammography. (A)** Screening is ineffective. **(B)** Screening is effective. **(C)** Screening leads to overdiagnosis. **(D)** Screening leads to overdiagnosis that causes death from side effects of treatment.

Mammography screening inevitably entails increased breast cancer incidence [36] due to earlier detection of cancers that would otherwise have been diagnosed later in life and due to diagnosis of cancers that would not have been identified clinically in someone's remaining lifetime. The latter category is commonly referred to as overdiagnosis. Theoretically, overdiagnosis can occur because the tumor lacks potential to progress to a clinical stage, or even regresses [37], or because the woman dies from other causes before the breast cancer surfaces clinically. In reality, these three alternatives cannot be reliably disentangled. In any of the three scenarios the individual woman would be diagnosed and treated with no possible survival benefit. Hence, overdiagnosis represents a substantial ethical dilemma and burdens the patient and the health care system. Treatment for breast cancer includes surgery, radiotherapy, chemotherapy, and antiestrogen treatment. Risk of death from cardiovascular disease is increased in women treated with radiotherapy [38], and adjuvant treatment may be cardiotoxic (for example, taxanes, anthracyclines, or trastuzumab) [39]. It is possible that overtreatment causes increased mortality by other causes besides breast cancer. This may explain why there is no reduction in measurable overall mortality with screening mammography [9] (Figure 2).

Overdiagnosis does apply to both carcinoma *in situ* and invasive cancer; the lifetime risk of progression of carcinoma *in situ* to invasive breast cancer is unknown, but probably less than 50%; [40] and the lead-time is longer for *in situ* than invasive cancers. Thus, it is logical and intuitive that carcinoma *in situ* can be overdiagnosed. However, pathological verified invasive cancers can also be overdiagnosed. This contradicts what most clinicians were taught in medical school, and can be hard to understand for both clinicians and the public. One way of looking at this challenge is by using the 'iceberg model' [40]: the development of cancer is a lengthy and complex process, where unrepaired genetic instability and changes in tumor microenvironment could lead to distinct, heterogeneous subpopulations of abnormal cells. Cancer can be envisioned as an iceberg of disease, where the visible tip above the waterline comprises the most aggressive lesions - those that produce symptoms and clinical disease. The majority of our body of knowledge concerning the natural history of malignancies comes from observations from these 'top-of-the-iceberg', symptomatic lesions above the waterline [40]. Underneath the water's surface, however, there might be multiple, indolent cancer subpopulations of cells. These subpopulations will look like cancer to the pathologist if detected through screening [40]. Early detection (such as mammography screening) dives under the surface and picks up silent lesions. The natural history of these asymptomatic lesions has not been studied and is therefore essentially unknown, but many of these may be indolent over time and never generate symptoms or disease without screening.

### Estimates of overdiagnosis

Precise estimation of overdiagnosis is a complicated and difficult task. There is no perfect analysis that would be universally applicable to this problem. Consequently, recent studies show a large variation in the estimated overdiagnosis of breast cancer, from none to 54% [41]. In studies based on statistical modeling to adjust for lead-time, estimates of overdiagnosis are consistently below 5% [42,43]. In contrast, observational studies have published higher estimates, between 22 and 54% [37,41,42], depending on the use of the denominator [44]. In Table 2, we present the amount of overdiagnosis and reduction in mortality estimated with different denominators (incidence/death from breast cancer in different age groups). It clearly shows that different denominators (rows 2 to 4 in Table 2) result in different amounts of overdiagnosis and mortality reduction. Thus, it is important that benefits and harms of mammography screening are presented using similar denominators (in Table 2).

**Table 2:** **Different percentages of overdiagnosis and mortality reduction based on the number of cancers overdiagnosed and deaths avoided from breast cancer using different denominators (incidence/death from breast cancer in different age-groups) in Norway in 2010**

**Age (years)**	**Expected number of cancers**	**Percentage of overdiagnosis (n = 714.4)**	**Expected number of breast cancer deaths**	**Percentage of mortality reduction (n = 53.7)**
50-99	2,208	19.4	693	7.8
50-79	1,571	27.3	506	10.6
50-69	942	45.5	334	16.1

The expected number of breast cancers and breast cancer deaths is estimated as the observed incidence and mortality rates in Norway from 1980 to 1984 multiplied by the Norwegian female population in 2010 [45-47]. The number of overdiagnosed cancers (714.4 cancers) is based on studies by Falk and coworkers [45] and Kalager and coworkers [44] and the number of reduced breast cancers (53.7 avoided deaths from breast cancer) is estimated by reducing the number of expected (358) breast cancer deaths by 15% in the age group 55 to 74 years (358 × 0.15 = 53.7).

Overdiagnosis might be underestimated in the statistical modeling studies because they tested only one assumption at a time, based either on assumptions for the risk of progression from carcinoma *in situ* to invasive cancer [42], or on sojourn time with adjustment for lead-time [42,43]. In statistical models based on sojourn time and lead-time, overdiagnosis has been disregarded in the estimation of lead-time, since the assumption of growth has been based on a progressive disease. This, however, is not the case for overdiagnosis where the disease is non-progressive or perhaps even regressive [37]. Thus, when using these estimates, overdiagnosis is likely to be underestimated [48].

Since we do not have any direct, biological evidence of non-progression or regression of breast cancer, assumptions cannot easily be tested, and represent only a 'guess'. Evidence from observational studies is more convincing. The difference in the estimates from observational studies (22 to 54%) might be due to different assumptions of expected changes in breast cancer incidence due to changes in breast cancer risk factors, different follow-up time after introduction of screening, and differences in accounting for lead-time. After 25 years of follow-up, the Canadian National Breast Screening Study [10], comparing physical breast examination with combined physical breast examination and annual mammography in women aged 40 to 59 years, found an excess of invasive cancer in the screening arm, resulting in 22% overdiagnosis. When the number of breast cancers detected at screening is used as the denominator (as in the Canadian study), the amount of overdiagnosis observed in the previous randomized trials is strikingly similar (22 to 24%) [10,49] and in line with the 30% reported in the Cochrane review of screening for breast cancer with mammography [9]. The amount of overdiagnosis might even be higher because DCIS, which accounts for one out of four breast cancers detected at mammography screening, was not included in these estimates [10]. If DCIS is a precursor of invasive breast cancer, we would expect a drop in incidence of invasive breast cancer after detection and removal of DCIS. There is no evidence for this. On the contrary, incidence rates keep increasing in countries with mammography screening [50].

**Figure 3:**
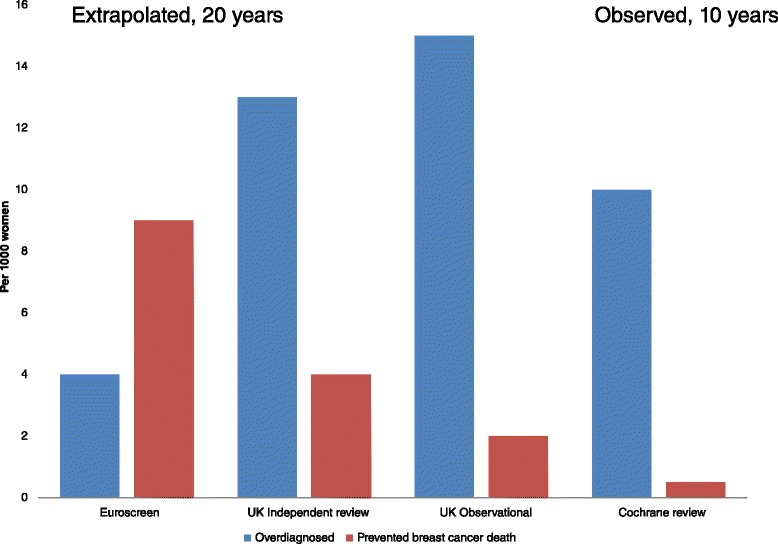
**Different estimates of overdiagnosed women and saved lives from breast cancer in different meta-analyses and trials.** Euroscreen: estimates derived from a review of observational studies, where estimates of mortality reduction from case–control studies are included [32]. UK Independent review: estimates on relative effect derived from randomized trials of mammography screening and applied to UK national rates for women aged 55 to 79 years [22]. UK Observational: estimates based on 31% overdiagnosis [19] and 13 to 17% reduction in mortality from breast cancer [35] and applied to the observed incidence of invasive breast cancer (women aged 50 to 69 years) and mortality (women aged 55 to 74 years) in the UK in 2007 [34]; this resulted in 2 to 3 prevented deaths from breast cancer. Cochrane review: estimates from the randomized trials of mammography screening [9]. The Cochrane review does not assume the effect of mammography screening to last for 20 years as is assumed in the other estimates, but relates to what was observed in the randomized trials [9].

Given the uncertainty of the estimates from modeling and observational studies, we used the best available estimate of overdiagnosis from observational data from a US study where DCIS and invasive cancer were included, follow-up was more than 25 years after screening was initiated and no extensive untestable assumptions were made [19]. However, in the US there is no mammography screening program, and the rate of false positives is higher than in Europe and Australia. Thus, it might be possible that the amount of overdiagnosis differs between the US and Europe and Australia. Since none of the estimates of overdiagnosis from Europe or Australia were based on follow-up as long as in the US study, we choose to use the US estimate of 31% overdiagnosis (in line with what is observed in the randomized trials) [19]. We estimated the number of overdiagnosed women based on the observed incidence of invasive breast cancer in women aged 50 to 69 years in the UK in 2007 [19,34,49]. For 1,000 women invited to biennial mammography screening for 20 years from age 50, 15 will be overdiagnosed (Figure 1). Based on different meta-analyses and reviews of benefits and harms of mammography screening [9,22,32] and our best estimate [19,34,35], we present a figure showing the different estimates of overdiagnosis and prevented deaths from breast cancer (Figure 3).

To be able to differentiate between potential lethal and non-lethal cancers, experimental studies have to be performed, preferably as an interdisciplinary cooperation between the biomedical and clinical communities. First, however, one has to accept that overdiagnosis does occur, and perhaps also change the terminology of non-lethal cancer to 'IDLE tumor' (InDolent Lesions of Epithelial origin), as recently suggested [6].

## Breast cancer mortality

According to the randomized breast cancer screening trials, the relative reduction in mortality from breast cancer ranges between 15 and 25% [9,22,36,51] for women aged 50 to 69 years. The differences in these estimates are due to differences in inclusion of randomized trials in pooled estimates. For the 25% estimated reduction, mammography screening versus no-screening is compared; thus, the Canadian trial was not included because they compared physical breast examination to combined physical breast examination and annual mammography [10,36]. For the 15% estimated reduction, methodological limitations in some of the randomized trials was accounted for [9]; without this 'adjustment', a 20% reduction was found [9,22,52]. None of the randomized trials showed any effect on cancer mortality or all-cause mortality [9]. Given the number of women enrolled in the randomized trials (660,000) and a 20% reduction in breast cancer mortality, a 2% reduction in all-cause mortality should have been detectable [52]. The absence of a reduction in all-cause mortality indicates that women die of other diseases at about the same time in life with and without screening.

### Study designs

There are a number of methods to investigate the effect of mammography screening in a non-experimental setting. Cohort studies, case–control studies, and trend studies show different estimates of mortality reduction, ranging from no effect to 50% reduction in breast cancer mortality [53,54].

#### Cohort studies

The optimal non-experimental design to investigate the effect of mammography screening is a cohort study of women invited and women not-invited to mammography screening who have similar baseline risk for breast cancer and breast cancer death and similar opportunities for optimal breast cancer treatment. Only few such studies exist, and the estimated effect of mammography screening on breast cancer mortality varies from 10 to 25% reduction [35]. A pooled estimate of these trials showed a reduction in breast cancer mortality of 13 to 17% [35].

#### Case–control studies

In case–control studies (sometimes called case-referent studies) cases are women who die of breast cancer and controls are women who are alive stratified by whether they have undergone screening mammography or not. Thus, these studies when performed in settings where mammography screening is recommended or where screening programs exist are comparisons of women who participate and who do not participate in mammography screening. The validity of these studies is low because of healthy screenee and self-selection bias, as women with breast cancer are not eligible to mammography screening or to be continued to be screened (selection of the most healthy), and women who choose to participate in mammography screening (selection) may differ with regard to risk of death from those who do not participate [55]. Attempts to adjust for these biases have been done by adjusting for the relative risk in breast cancer mortality between the non-participants and the non-invited comparison group [7,56]. The underlying assumption of these adjustments is that we do know the risk of uninvited women. In randomized trials, we can easily find the risk of breast cancer death for those not invited to mammography screening (the control group). However, in observational studies where everybody is invited or recommended to undergo mammography screening, we have to make assumptions on risk of death from breast cancer among the uninvited women. These assumptions cannot be tested and are therefore based on 'best guess' estimates. In case–control studies, a 50% reduction in mortality from breast cancer is found, and similar reductions are found in cohort studies of participants and non-participants in mammography screening [54,57]. When the randomized trial from Malmö was analyzed as a case–control study, a 58% reduction in mortality from breast cancer was found, whereas the real, observed reduction in the trial was only 4% (8% when the results were adjusted for non-compliance and contamination) [36]. Thus, estimates from case–control studies systematically overestimate the effect of screening.

#### Trend studies

Trend studies are studies of population-based breast cancer mortality over time in different ages (age-standardization) and geographic areas. Data on population-based breast cancer mortality are easy to retrieve, but as the yearly mortality rate is not reflective of time of diagnosis, deaths from breast cancer diagnosed before invitation influences the mortality rate some years after screening is implemented. Further, when all eligible women are invited and a screening program has been running for some time, the mortality rate is expected to reach a steady state and further reduction cannot be expected. After 7 years of follow-up in the Health Insurance Plan study, the mortality reduction was no longer apparent [58], indicating that screening has no effect if no longer offered. For a continuing program, however, the mortality effect will not disappear, but reach a steady state. Thus, in the first years after screening has been introduced and reached full coverage in the area studied, the cause of change in trends of breast cancer mortality can be difficult to study and interpret. Most trend studies show that breast cancer mortality has declined in most European countries since the early to mid-1990s. The decline in mortality is even higher among women younger than the eligible age range for screening and for some countries a reduction is observed also for women older than the eligible age range [59]. The interpretation of these results could be that heightened awareness and improved therapy rather than mammography screening are responsible for the observed reduction [53,59,60].

### Tumor stage

Another benefit of mammography screening could be that breast cancers detected at screening are smaller and thus less advanced than those detected clinically. In general, smaller tumors are more likely to be resected by lumpectomy, and with less node-positive disease, less adjuvant therapy is needed. Based on the randomized mammography screening trials, however, this is not the case; screening was associated with an increase in the number of mastectomies of about 20% [9]. The reason is that mammography increased both the number of women diagnosed with invasive breast cancer and the number found to have multiple microscopic cancers distributed throughout the breast, for which mastectomy is recommended. Further, in the National Health Service breast screening program in the UK, 30% of DCIS and 24% of invasive breast cancers were treated with mastectomy, so earlier detection does not necessarily mean less aggressive treatment [61]. As mentioned above, another benefit of mammography screening could be less aggressive adjuvant therapy, due to smaller and less aggressive tumors. As seen in the stage distribution in screening and non-screening groups in Norway [41], screening led to the diagnosis of 58% more stage I (localized cancer) and 22% more stage II (regional cancer or cancer involving the lymph nodes) cancers, without any reduction in advanced stage disease (stages III and IV). Since all these patients receive surgery (either mastectomy or breast-conserving surgery with radiation) and most stage II patients are recommended to receive adjuvant chemotherapy, screening may have led to 58% more women undergoing breast surgery and 22% more women undergoing adjuvant chemotherapy [41]. Thus, screening mammography does not seem to reduce the burden of receiving more aggressive treatment.

### Cause of death

**Figure 4:**
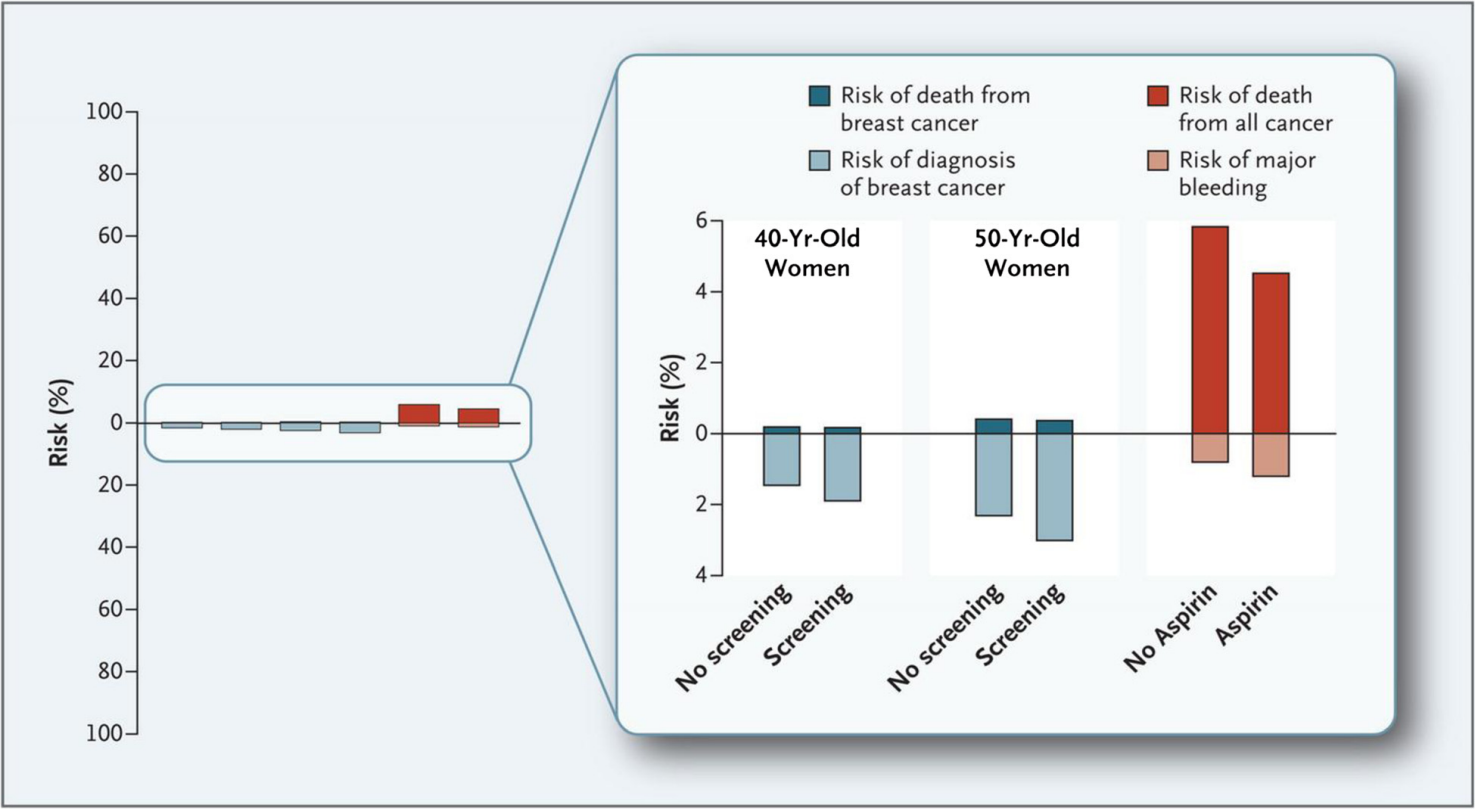
**Benefit and harm with screening mammography and use of aspirin over 10 years** **[** 62 **]** **.** Shown are the 10-year risk of death from breast cancer (bars above 0) and the 10-year risk of the diagnosis of breast cancer (bars below 0) among women aged 40 years and 50 years, with and without mammography screening. Also shown are the 10-year risk of death from cancer (bar above 0) and the 10-year risk of major extracranial bleeding, defined as bleeding necessitating transfusion or resulting in death (bar below 0), associated with the use or non-use of aspirin as a primary preventive measure (on the basis of findings from randomized trials). In each pair (no screening versus screening and no aspirin versus aspirin), the difference between the percentages represented by the bars shows the absolute benefit or harm associated with screening mammography or the use of aspirin. Background data are derived from the literature.

**Figure 5:**
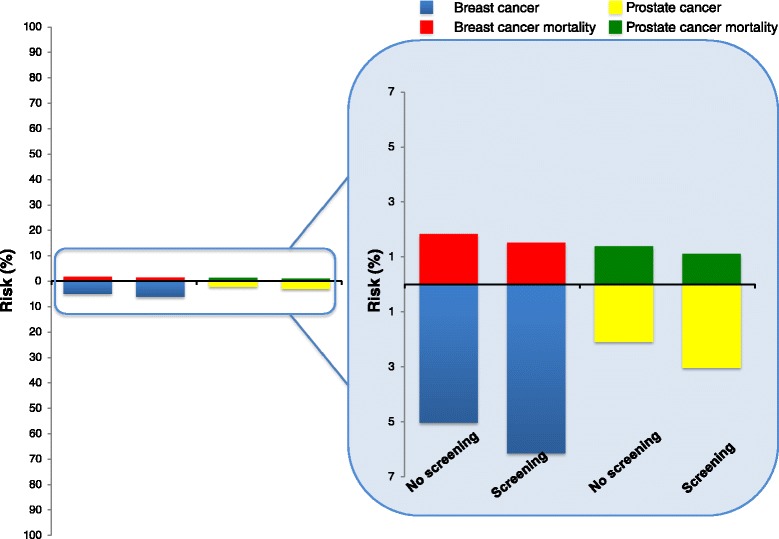
**Twenty year risk for diagnosis of, and death from, breast and prostate cancer with and without screening in the United Kingdom** **[** 49 **]** **.** Displayed are 20-year absolute risks for incidence (including overdiagnosis) and mortality with and without screening. Overdiagnosis is set to 45% for prostate cancer and 22% for breast cancer, respectively (age 50 to 69 years). Mortality reduction is set to be 20% for both cancers (age 55 and 74 years). For prostate cancer, the estimates are based on the observed incidence and mortality in 1998 (before any widespread use of prostate-specific antigen (PSA)) and for breast cancer in 2007 (latest data available).

The number of women saved from breast cancer death might be outweighed by death from other causes due to harms of treatment; however, due to uncertainty about the overall number of women saved, we present different estimates of women saved from breast cancer in different meta-analyses of randomized and observational studies of breast cancer [19,22,32,34,35] (Figure 3). The number needed to be invited to mammography to save or harm women is highly dependent on the underlying risk of breast cancer or death from breast cancer (Figures 4 and 5, showing risk of breast cancer and death from breast cancer in the US and UK [49,62]). In the estimates shown in Figure 1, we use UK data from 2007 for mortality from breast cancer in women aged 55 to 74 years [34], and the relative reduction of 13 to 17% in breast cancer mortality based on a meta-analysis of observational studies [35]. For 1,000 women invited to mammography screening every second year for 20 twenty years from age 50, 2 to 3 women are prevented from dying from breast cancer (Figure 1).

## Information to women

Screening differs from clinical practice. Individuals who undergo a screening procedure are invited to participate with the implied expectation that they will benefit. This contrasts with clinical practice, where the patients approach the medical practitioner with a symptom or complaint for help [3]. Thus, it is of utmost importance that information about benefits and harms of mammography screening is balanced. However, the harms of screening have not been communicated to the public as well as the benefits [63,64]. With increasing evidence of overdiagnosis, this is of concern and violates the individual's possibility to make an informed choice.

However, proper information on risks and benefits is not easy. Firstly, how do clinicians communicate benefits and harms? The use of relative risks may suggest greater effects than exist, whereas the use of absolute risks (or equivalents, such as the number needed to screen) prevents this misunderstanding. The use of relative risks should be avoided or employed only in combination with more comprehensible forms of communicating risk, such as absolute risks or numbers needed to screen [65]. Secondly, many cannot interpret numbers as well as words and have difficulty understanding numerical expressions of risk [66]. In medical schools, courses in statistics usually do not go far enough in teaching statistical or probabilistic thinking, and few teach strategies for effective communication. Hence, most physicians are poorly equipped to discuss risk factors in a way that is readily comprehensible to their patients. This deficiency puts the ideal of informed consent in jeopardy [65,67].

**Figure 6:**
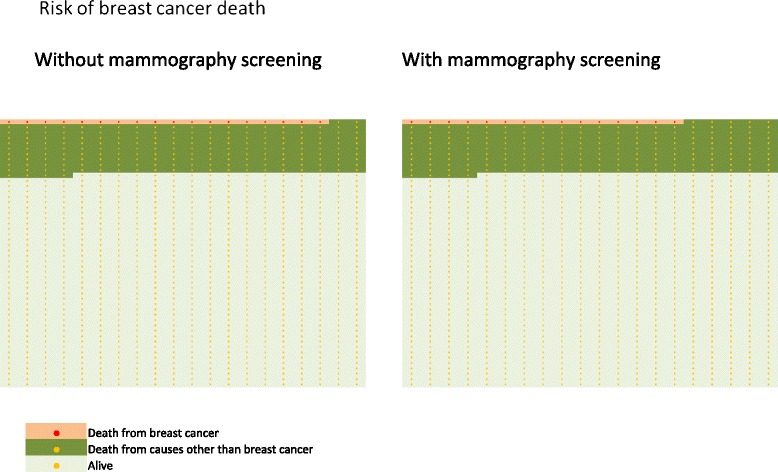
**Positive framing.** Out of 1,000 women aged 50 to 69 years invited every second year, 781 are alive with screening and the same number without screening over the course of 20 years. Correspondingly, 985 women and 982 to 983 women without screening will not die of breast cancer aged 55 to 74 years. Negative framing: out of 1,000 women aged 50 to 69 years invited every second year, 204 women will die with screening and the same number without screening. Correspondingly, 15 women with screening and 17 to 18 women without screening will die of breast cancer between 55 and 74 years old. Number of women dying among women aged 55 to 74 years is based on the observed mortality rates in England and Wales in 2007 [68]. The number of women dying over a 20-year period is estimated by summing the mortality rates for the ages 55 to 74 [68].

Framing is the presentation of logically equivalent information in different forms. Positive framing emphasizes the absence of disease; negative framing emphasizes the presence of disease [65] (Figure 6). Based on the 20-year risk for a woman in the UK to die of breast cancer, the risk of dying from breast cancer with mammography screening would be 15 per 1,000 women and 17 to 18 per 1,000 women without mammography screening [49]. Positive framing would be that the number of women that will not die from breast cancer rises from between 982 and 983 to 985 per 1,000 women with the addition of screening for breast cancer [34,35]. An example of positive framing is illustrated in Figure 6.

Women are not only overestimating their risk of breast cancer, but also substantially overestimating the benefit of mammography screening [67,69-71]. Over 50% of all women asked thought mammography screening reduced the risk of dying from breast cancer by at least 50% [67,69]. Further, women wanted to have balanced information and share the decision with their physician [71], but many reported they were never provided information on false positives and side effects [71]. A report from Norway, where women are invited with a prescheduled time and date of a screening mammography appointment, showed that if the invitation letter included an information leaflet aimed at enabling women to make a free and informed choice, the prescheduled appointment undermined the option of not participating [72]. The authors concluded that the current recruitment procedures gave priority to screening uptake at the expense of informed choice [72]. Thus, the principle of informed choice might be in jeopardy [72].

## Conclusion

Women should be correctly informed about the benefits and harms of mammography screening (Figures 1 and 2). A comprehensible way of communicating information on benefits and harms of mammography screening is presented in Figure 1: among 1,000 women who start screening at age 50 and are screened for 20 years, 2 to 3 will avoid dying from breast cancer and 200 women will have at least one false positive test, 30 will undergo a biopsy, 3 will be diagnosed with an interval cancer, and breast cancer will be overdiagnosed in 15.

In an era of limited resources for health care and preventive services, we need to scrutinize our efforts in screening and prevention. One of the overarching goals of screening is the reduction of incidence or mortality of disease. Currently, we do recommend some screening services (such as mammography), while others are debated or discouraged (such as prostate-specific antigen screening for prostate cancer or aspirin for primary prevention of cardiovascular disease and premature death). However, as Figures 4 and 5 show, these differences in recommendations do often not reflect differences in effectiveness or harms between the different tests [49,62].

## Abbreviation

DCIS: Ductal carcinoma *in situ*

## References

[1] Shorter Oxford English Dictionary. Oxford, United Kingdom: Oxford University Press; 2010

[2] Raffle AE, Gray JAM (2007). Screening: Evidence and Practice.

[3] Holland WW, Stewart S (2005). Screening in Disease Prevention. What works?.

[4] Bretthauer M, Kalager M (2013). Principles, effectiveness and caveats in screening for cancer. Br J Surg.

[5] Wilson JMG, Junger G (1968). Principles and Practice of Screening for Disease.

[6] Esserman L, Thompson IM, Reid B, Nelson P, Ransohoff DF, Welch HG (2014). Addressing overdiagnosis and overtreatment in cancer: a prescription for change. Lancet Oncol.

[7] Aronowitz RA (2007). Unnatural History: Breast Cancer and American Society.

[8] Reynolds H (2012). The Big Squeeze: a Social and Political History of the Controversial Mammogram.

[9] Gøtzsche P, Jørgensen KJ (2013). Screening for breast cancer with mammography. Cochrane Database Syst Rev.

[10] Miller A, Wall C, Bains C, Sun P, To T, Narod S (2014). Twenty-five year follow-up of the Canadian national breast screening study. BMJ.

[11] Moss SM, Cuckle H, Evans A, Johns L, Waller M, Bobrow L (2006). Effect of mammographic screening from age 40 years on breast cancer mortality at 10 years' follow-up: a randomised controlled trial. Lancet.

[12] Gøtzsch P, Olsen O (2000). Is screening for breast cancer with mammography justified?. Lancet.

[13] Jørgensen KJ, Keen JD, Gøtzcshe PC (2011). Is mammographic screening justifiable considering its substantial overdiagnosis rate and minor effect on mortality?. Radiology.

[14] Kopans DB, Smith RA, Duffy SW (2011). Mammographic screening and “overdiagnosis”. Radiology.

[15] Mandelblatt JS, Cronin KA, Bailey S, Berry DA, de Koning HJ, Draisma G (2009). Effects of mammography screening under different screening schedules: model estimates of potential benefits and harms. Ann Intern Med.

[16] Nelson HD, Tyne K, Naik A, Bougatsos C, Chan BK, Humphrey L, US Preventive Services Task Force (2009). Screening for breast cancer: an update for the U.S. Preventive Services Task Force. Ann Intern Med.

[17] Swiss Medical Board. Systematisches Mammographie-Screening [http://www.medical-board.ch/fileadmin/docs/public/mb/Fachberichte/2013-12-15_Bericht_Mammographie_Final_rev.pdf]

[18] Giordano L, von Karsa L, Tomatis M, Majek O, de Wolf C, Lancucki L (2012). Mammographic screening programmes in Europe: organization, coverage and participation. J Med Screen.

[19] Bleyer A, Welch H (2012). Effect of three decades of screening mammography on breast-cancer incidence. N Engl J Med.

[20] Törnberg S, Kemetli L, Ascunce N, Hofvind S, Anttila A, Sèradour B (2010). A pooled analysis of interval cancer rates in six European countries. Eur J Cancer Prev.

[21] Hofvind S, Ponti A, Patnick J, Ascunce N, Njor S, Broeders M (2012). False-positive results in mammographic screening for breast cancer in Europe: a literature review and survey of service screening programmes. J Med Screen.

[22] Independent UK Panel on Breast Cancer Screening (2012). The benefits and harms of breast cancer screening: an independent review. Lancet.

[23] Elmore JG, Barton MB, Moceri VM, Polk S, Arena PJ, Fletcher SW (1998). Ten-year risk of false positive screening mammograms and clinical breast examinations. N Engl J Med.

[24] Hubbard RA, Kerlikowske K, Flowers CI, Yankaskas BC, Zhu W, Miglioretti DL (2011). Cumulative probability of false-positive recall or biopsy recommendation after 10 years of screening mammography: a cohort study. Ann Intern Med.

[25] Lidbrink E, Elfving J, Frisell J, Jonsson E (1996). Neglected aspects of false positive findings of mammography in breast cancer screening: analysis of false positive cases from the Stockholm trial. BMJ.

[26] Brodersen J, Siersma VD (2013). Long-term psychosocial consequences of false-positive screening mammography. Ann Fam Med.

[27] van der Steeg AFW, Keyzer-Dekker CM, De Vries J, Roukema JA (2011). Effect of abnormal screening mammogram on quality of life. Br J Surg.

[28] Barton M (2001). Increased patient concern after false-positive. J Gen Intern Med.

[29] Kalager M, Tamimi R, Bretthauer M, Adami H-O (2012). Prognosis in women with interval breast cancer: population based observational cohort study. BMJ.

[30] Hofvind S, Skaane P, Vitak B, Wang H, Thoresen S, Eriksen L (2005). Influence of review design on percentages of missed interval breast cancers: a retrospect study of interval cancers in a population-based screening program. Radiology.

[31] Hoff SR, Abrahamsen AL, Samset JH, Vigeland E, Klepp O, Hofvind S (2012). Breast cancer: missed interval and screening-detected cancer at full-field digital mammography and screen-film mammography - results from a retrospective review. Radiology.

[32] Paci E, EUROSCREEN Working Group (2012). Summary of the evidence of breast cancer service screening outcomes in Europe and first estimate of the benefit and harm balance sheet. J Med Screen.

[33] NHS Breast Screening Programme. National collation of breast interval cancer data: Screening years 1st April 2003-31st March 2005 [http://www.cancerscreening.nhs.uk/breastscreen/publications/nhsbsp-occasional-report1203.pdf]

[34] Steliarova-Foucher E, O'Callaghan M, Ferlay J, Masuyer E, Forman D, Comber H, et al. European Cancer Observatory: Cancer Incidence, Mortality, Prevalence and Survival in Europe. Version 1.0 (September 2012). European Network of Cancer Registries, International Agency for Research on Cancer [http://eco.iarc.fr, http://eco.iarc.fr/EUREG/AnalysisG.aspx]

[35] Irvin VL, Kaplan RM (2014). Screening mammography & breast cancer mortality: meta-analysis of quasi-experimental studies. PLoS One.

[36] Vainio H, Bianchini F (2002). IARC Handbook of Cancer Prevention. Volume 7. Breast Cancer Screening.

[37] Zahl PH, Mælen J, Welch HG (2008). The natural history of invasive breast cancers detected by screening mammography. Arch Intern Med.

[38] Darby SC, Ewertz M, McGale P, Bennet AM, Blom-Goldman U, Brønnum D (2013). Risk of ischemic heart disease in women after radiotherapy for breast cancer. N Engl J Med.

[39] Yeh ETH, Bickford C (2009). Cardiovascular complications of cancer therapy: incidence, pathogenesis, diagnosis, and management. J Am Coll Cardiol.

[40] Kramer BS, Croswell JM (2009). Cancer screening: the clash of science and intuition. Annu Rev Med.

[41] Kalager M, Adami H-O, Bretthauer M, Tamimi RM (2012). Overdiagnosis of invasive breast cancer due to mammography screening: results from the Norwegian Screening Program. Ann Intern Med.

[42] Puliti D, Duffy SW, Miccinesi G, deKoning H, Lynge E, Zappa M (2012). Overdiagnosis in mammographic screening for breast cancer in Europe: a literature review. J Med Screen.

[43] de Koning HJ, Draisma G, Francheboud J, de Bruijn A (2006). Microsimulation modeling estimates based on observed screen and clinical data. Breast Cancer Res.

[44] Kalager M, Løberg M, Fønnebø VM, Bretthauer M (2013). Failure to account for selection-bias. Int J Cancer.

[45] Falk RS, Hofvind S, Skaane P, Haldorsen T (2013). Overdiagnosis among women attending a population-based mammography screening program. Int J Cancer.

[46] Statistics Norway. Population, by sex and age [https://www.ssb.no/statistikkbanken/selectvarval/Define.asp?subjectcode%09=%09&ProductId%09=%09&MainTable%09=%09FolkemEttAarig&nvl%09=%09&PLanguage%09=%090&nyTmpVar%09=%09true&CMSSubjectArea%09=%09befolkning&KortNavnWeb%09=%09folkemengde&StatVariant%09=%09&checked%09=%09true&checked=true]

[47] NORDCAN Database [http://www-dep.iarc.fr/NORDCAN/english/frame.asp]

[48] Zahl PH, Jørgensen KJ, Gøtzsche P. Lead-time models should not be used to estimate overdiagnosis in cancer screening. J Gen Intern Med. 2014;doi:10.007/s11606-014-2812-2. 10.1007/s11606-014-2812-2 PMC4139512 24590736

[49] Kalager M, Adami HO, Bretthauer M (2014). Too much mammography. BMJ.

[50] Jørgensen KJ, Gøtzsche PC (2009). Overdiagnosis in publicly organized mammography screening programmes: systematic review of incidence trends. BMJ.

[51] Humphrey LL, Helfand M, Chan BK, Woolf SH (2002). Breast cancer screening: a summary of the evidence for the U.S. Preventive Services Task Force. Ann Intern Med.

[52] Jüni P, Zwahlen M (2014). It is time to initiate another breast cancer screening trial. Ann Intern Med.

[53] Jørgensen KJ, Zahl PH, Gøtzsche PC (2010). Breast cancer mortality in organized mammography screening in Denmark: comparative study. BMJ.

[54] Nickson C, Mason KE, English DR, Kavanagh AM (2012). Mammographic screening and breast cancer mortality: a case–control study and meta-analysis. Cancer Epidemiol Biomarkers Prev.

[55] Morrison AS (1992). Screening in Chronic Disease.

[56] Duffy SW, Cuzick J, Tabar L, Vitak B, Chen THH, Yen MF (2002). Correcting for non-compliance bias in case–control studies to evaluate cancer screening programmes. Appl Stat.

[57] Hofvind S, Ursin G, Tretli S, Sebuødegård S, Møller B (2013). Breast cancer mortality in participants of the Norwegian Breast Cancer Screening Program. Cancer.

[58] Shapiro S (1997). Screening for breast cancer: the HIP Randomized Controlled Trial. Health Insurance Plan. J Natl Cancer Inst Monogr.

[59] Autier P, Boniol M, LaVecchia C, Vatten L, Gavin A, Héry C (2010). Disparities in breast cancer mortality trends between 30 European countries: retrospective trend analysis of WHO mortality database. BMJ.

[60] Autier P, Boniol M, Gavin A, Vatten LJ (2011). Breast cancer mortality in neighbouring European countries with different levels of screening but similar access to treatment: trend analysis of WHO mortality database. BMJ.

[61] NHS Breast Screening Programme and Association of Breast Surgery. An Audit of Screen Detected Breast Cancers for the Year of Screening April 2010 to March 2011 [http://www.cancerscreening.nhs.uk/breastscreen/publications/baso2010-2011.pdf]

[62] Smith RA, Kerlikowske K, Miglioretti DL, Kalager M (2012). Principles, effectiveness and caveats in screening for cancer. N Engl J Med.

[63] Jørgensen KJ, Gøtzsche PC (2006). Content of invitation for publicly funded screening mammography. BMJ.

[64] Jørgensen KJ, Gøtzsche PC (2004). Information in practice. Presentation on websites of possible benefits for breast cancer: cross sectional study. BMJ.

[65] Elmore JG, Gigerenzer G (2005). Benign breast disease- the risk of communicating risk. N Engl J Med.

[66] Deyo RA, Patrick DL (2005). Hope or Hype: the Obsession with Medical Advances and the High Cost of False Promises.

[67] Chamot E, Perneger TV (2001). Misconceptions about efficacy of mammography screening: a public health dilemma. J Epidemiol Community Health.

[68] Office for National Statistics. Mortality statistics: death registered in 2007 [http://www.ons.gov.uk/ons/datasets-and-tables/index.html?newquery%09=%09mortality%09+%09statistics%3Adeath%09+%09registered%09+%09in%09+%092007&newoffset%09=%09150&pageSize%09=%0950&content-type%09=%09Reference%09+%09table&content-type%09=%09Dataset&content-type-orig%09=%09%22Dataset%22%09+%09OR%09+%09content-type_original%3A%22Reference%09+%09table%22&sortBy%09=%09none&sortDirection%09=%09none&applyFilters%09=%09true]

[69] Domenighetti G, D'Avanzo B, Egger M, Berrino F, Perneger T, Mosconi P (2003). Women’s perception of the benefits of mammography screening: population based survey in four countries. Int J Epidemiol.

[70] McMenamin M, Barry H, Lennon AM, Purcell H, Baum M, Keegan D (2005). A survey of breast cancer awareness and knowledge in a Western population: lots of light but little illumination. Eur J Cancer.

[71] Davey HM, Barratt AL, Davey E, Butow PN, Redman S, Houssami N (2002). Medical tests: women’s reported and preferred decision-making roles and preferences for information on benefits, side-effects and false results. Health Expect.

[72] Østerlie W, Solbjør M, Skolbekken JA, Hofvind S, Sætnan AR, Forsmo S (2008). Challenges of informed choice in organized screening. J Med Ethics.

